# Circulating NETosis biomarkers predict severity, 12-month functional outcome, and survival after spontaneous intracerebral hemorrhage

**DOI:** 10.3389/fneur.2026.1901302

**Published:** 2026-09-14

**Authors:** Chaoshuai Hu, Chi Ma, Ming Yang, Fan Wang, Shifang Zhou, Lei Hui

**Affiliations:** 1 Department of Neurosurgery, The First Affiliated Hospital of Henan Medical University, Weihui, China; 2 Department of Neurology, The First Affiliated Hospital of Henan Medical University, Weihui, China

**Keywords:** cell-free DNA, citrullinated histone H3, intracerebral hemorrhage, NEtosis, nucleosome, prognosis

## Abstract

**Background:**

Spontaneous intracerebral hemorrhage (ICH) carries high early mortality and long-term disability; however, circulating biomarkers that capture both bleeding severity and prognosis are lacking. Neutrophil extracellular trap formation (NETosis), quantifiable in plasma as citrullinated histone H3 (H3Cit), cell-free DNA (cfDNA), and nucleosomes, is implicated in secondary brain injury after ICH.

**Methods:**

Three hundred fifteen adults with first-ever spontaneous ICH within 24 h of onset and 160 community controls were enrolled. Plasma H3Cit, cfDNA and nucleosomes, admission hematoma volume, NIHSS, and GCS were recorded; the modified Rankin Scale (mRS) was assessed at 12 months. NETosis biomarkers entered linear, logistic, and Cox regressions as log2-transformed continuous variables and were adjusted progressively for time-to-sampling, demographics, vascular risk factors, surgical treatment, NIHSS, and hematoma volume. Incremental predictive value over a clinical baseline model was assessed using area under the receiver operating characteristic curve (AUC)/concordance index (C-index), continuous net reclassification improvement (NRI), and integrated discrimination improvement (IDI).

**Results:**

All three biomarkers were higher in ICH than controls (all *p* < 0.001) and were several-fold higher in 12-month decedents than survivors, despite no difference in absolute neutrophil count. After adjustment, H3Cit was independently associated with hematoma volume, NIHSS and GCS; cfDNA and nucleosomes were independently associated with hematoma volume. After full adjustment for demographics, vascular risk factors, surgical treatment, NIHSS, and hematoma volume, each doubling of H3Cit (OR: 1.577, 95% CI: 1.368–1.838), cfDNA (OR: 1.687, 95% CI: 1.259–2.291), and nucleosomes (OR: 1.622, 95% CI: 1.297–2.057) independently predicted 12-month mRS ≥ 3 (all *p* < 0.001); per doubling, H3Cit (HR: 1.358, 95% CI: 1.207–1.528), cfDNA (HR: 1.619, 95% CI: 1.287–2.038), and nucleosomes (HR: 1.568, 95% CI: 1.310–1.877) independently predicted 12-month death. Adding H3Cit raised the AUC for mRS ≥ 3 from 0.814 to 0.869 (ΔAUC: 0.055, *p* < 0.001) and the C-index for death from 0.765 to 0.818 (ΔC: 0.052, *p* < 0.001); for cfDNA and nucleosomes, the gains in discrimination were not significant, although NRI and IDI improved.

**Conclusion:**

Plasma H3Cit, cfDNA, and nucleosomes within 24 h of ICH track hemorrhage severity and predict 12-month mRS ≥ 3 and death beyond established clinical scores, with H3Cit showing the most consistent incremental value.

## 1 Introduction

Spontaneous ICH is the most lethal acute stroke subtype, with roughly half of patients dying within 1 year and a large fraction of survivors left with substantial dependence (1, 2). Global epidemiological data continue to position ICH as a leading cause of stroke-related death and disability worldwide, with rising absolute burden in aging populations (3, 4). The outcomes are driven not only by the primary hematoma but also by hematoma expansion, perihematomal edema, and a complex cascade of secondary injury comprising oxidative stress, iron toxicity, blood–brain barrier breakdown, and intense neuroinflammation (5–7). Inflammatory responses, in particular the early infiltration of peripheral neutrophils, contribute to brain edema, white matter injury, and long-term disability after ICH (8–10). Despite recent progress in surgical and supportive strategies, no targeted disease-modifying therapy for ICH exists, and prognostic decisions still rely heavily on initial hematoma volume, the National Institutes of Health Stroke Scale (NIHSS), and the Glasgow Coma Scale (GCS) (11–13).

A wide range of circulating biomarkers has been explored to refine prognostication after ICH, including markers of axonal injury such as neurofilament light chain (NfL), acute-phase proteins (SERPINA3, C-reactive protein), inflammatory cytokines (interleukin-6), complement factors (C1q), glial proteins (S100B), and markers of stress hyperglycemia (14–18). However, each of these markers reports a different facet of the response: NfL primarily reflects late axonal damage rather than acute hemorrhagic burden (15), inflammatory cytokines such as IL-6 index the overall intensity of the downstream inflammatory response rather than the cellular process that generates it (17), and acute-phase reactants such as SERPINA3 capture systemic injury without resolving its cellular source (16). Anemia and other systemic responses further confound prognostic signals derived from broad inflammatory indices (19). A parallel and considerably more mature literature has established that indices of systemic immune activation derived from the routine hemogram—the neutrophil-to-lymphocyte ratio (NLR), the platelet-to-white-blood-cell ratio (PWR), and the systemic inflammation response index (SIRI)—carry genuine prognostic information after ICH, are inexpensive, and are readily available (20–22). These indices, however, quantify the number and relative composition of circulating leucocytes rather than the specific effector program those cells have executed, and the information they carry is not always interchangeable: In the same cohort, SIRI but not the closely related systemic immune-inflammation index was independently associated with in-hospital infection and 3-month outcome (22). Therefore, there is continued interest in biomarkers that more directly index the early, mechanistically relevant immune events after ICH.

NETosis is a regulated form of neutrophil cell death in which decondensed chromatin, citrullinated histones, and granular proteins are extruded into the extracellular space (23). NETs amplify thromboinflammation and engage the complement and coagulation systems, linking innate immunity to vascular injury (24). In ICH, neutrophils accumulate within and around the hematoma and release NETs that have been visualized in post-mortem human brain tissue and replicated in animal models (25–27). Mechanistically, NETs promote perihematomal edema through ERK-mediated regulation of MMP9 and AQP4 (28), impair meningeal lymphatic drainage to aggravate secondary hydrocephalus (29), and drive microglial activation and chronic neuroinflammation (30). Pre-clinical inhibition of NET formation attenuates brain injury in ICH and ischemic stroke models, supporting NETs as a therapeutic target as well as a biomarker substrate (31, 32). These observations make circulating NETosis indices a biologically motivated candidate biomarker class for ICH.

Three plasma analytes have emerged as practical readouts of NETosis: H3Cit, which is enzymatically generated by PAD4 and is the most specific of the three; cell-free DNA (cfDNA), which is more sensitive but also reflects other forms of cell death; and circulating nucleosomes, which capture chromatin release in NETs and other contexts (33–36). These NETosis biomarkers predict mortality in cancer, sepsis, and cardiovascular disease (33, 35, 36), but their joint association with hemorrhage burden, functional disability, and survival after ICH has not been systematically characterized.

Therefore, we conducted a cohort study to test whether circulating H3Cit, cfDNA, and nucleosomes measured within 24 h of symptom onset are associated with admission severity (hematoma volume, NIHSS, and GCS), 12-month functional outcome (mRS), and 12-month survival in adults with spontaneous ICH and to quantify their incremental predictive value beyond an established clinical baseline model.

## 2 Materials and methods

### 2.1 Study design and participants

This was a single-center observational cohort study conducted in the Department of Neurosurgery of the First Affiliated Hospital of Henan Medical University between January 2022 and January 2025. Consecutive adults admitted with spontaneous ICH were screened. The inclusion criteria were patients aged >18 years and first-ever ICH with blood collection within 24 h of symptom onset. The exclusion criteria were primary subarachnoid hemorrhage, hemorrhagic transformation of cerebral infarction, post-thrombolysis hemorrhage, traumatic ICH, isolated subdural or epidural hematoma, ICH attributable to other identifiable diseases, neurodegenerative disease, tumor, and overt cognitive impairment or dementia. A total of 315 patients were ultimately enrolled. Healthy community controls (*n* = 160) were recruited contemporaneously from routine medical-examination volunteers without history of stroke, cancer, active infection, or major systemic disease.

All participants provided written informed consent. The study was conducted in accordance with the Declaration of Helsinki and was approved by the Ethics Committee of The First Affiliated Hospital of Henan Medical University (approval no. EC-LW26-127).

### 2.2 Clinical assessment and follow-up

Demographics, vascular risk factors, and routine laboratory data were recorded at admission. Severity variables of pre-specified interest were admission hematoma volume, estimated by the ABC/2 method, the NIHSS score, and the GCS score, all recorded at the time of blood sampling. All ICH patients were followed for vital status at 1, 3, 6, and 12 months after discharge, and functional outcome was determined at 12 months at an outpatient clinic visit using the mRS and dichotomized as mRS < 3 (favorable) versus (vs.) mRS ≥ 3 (unfavorable).

### 2.3 NETosis biomarker measurement

Peripheral venous blood was collected at admission, before any surgical intervention and before the administration of perioperative medication, into sodium citrate tubes, centrifuged at 3000 × g for 10 min, and platelet-poor plasma was stored at −80 °C until batch analysis. Three NETosis indices were measured using the same assay panel previously described by Grilz et al. (33) In brief, plasma H3Cit was quantified by an in-house sandwich ELISA: 96-well plates (Nunc™ MicroWell™, Thermo Fisher Scientific) were coated with the anti-histone capture antibody from the Cell Death Detection ELISA Kit (Roche Diagnostics, Mannheim, Germany); plasma samples were applied and detected with a rabbit anti-citrullinated histone H3 antibody (ab5103, Abcam, Cambridge, MA, United States), followed by HRP-conjugated goat anti-rabbit IgG secondary antibody (Bio-Rad) and TMB substrate (Sigma-Aldrich) developed at 450 nm. The results are reported in ng/mL; samples whose absorbance fell below the blank were assigned an H3Cit value of 0 ng/mL, as in the validated assay (34). Plasma cfDNA was quantified using the Quant-iT PicoGreen dsDNA Assay Kit (Thermo Fisher Scientific) and expressed in ng/mL. Plasma nucleosomes were measured with the Cell Death Detection ELISAPLUS kit (Roche Diagnostics) and normalized to pooled plasma from young healthy male controls to yield a multiple of the median (MoM) value. All measurements were performed in duplicate by operators blinded to clinical data.

Assay performance was monitored with a pooled plasma quality-control (QC) sample prepared by combining equal volumes of 30 randomly selected study samples and mixing thoroughly; eight replicate QC wells were included on every 96-well plate. For each plate, the mean and standard deviation of the concentrations measured in its eight QC wells were calculated, and the intra-assay coefficient of variation (CV) for that plate was the standard deviation divided by the mean. The inter-assay CV was obtained by first calculating the mean QC concentration of each plate and then dividing the standard deviation of these plate-level QC means by their mean across all plates. For the in-house H3Cit sandwich ELISA, the highest intra-assay CV was 8.4% and the inter-assay CV was 12.8%; the corresponding values were 5.2 and 8.5% for the cfDNA PicoGreen assay and 7.1 and 11.3% for the nucleosome ELISA.

### 2.4 Statistical analysis

Normally distributed continuous variables are summarized as mean ± SD and non-normally distributed continuous variables as median (interquartile range); categorical variables are summarized as counts (percentages). Continuous variables were compared between the two groups with the independent *t*-test or the Mann–Whitney U test and across the three groups with the Kruskal–Wallis test; categorical variables were compared with the *χ*^2^ test or Fisher’s exact test. Correlations between continuous variables were assessed by Spearman’s correlation.

To stabilize variance and improve linearity, NETosis biomarkers entered all regression models as log2-transformed continuous variables, whereas admission hematoma volume, NIHSS, and GCS scores were modeled in linear regression as square-root-transformed variables, in line with the analytic templates established in prior ICH biomarker cohorts (15, 16). Three sets of regression analyses were performed:

- Multivariable linear regression separately assessed the association of each NETosis biomarker with √hematoma volume, √NIHSS, and √GCS. β coefficients and 95% CIs were estimated and interpreted as the change in mean √hematoma volume, √NIHSS, or √GCS for each doubling of the NETosis biomarker concentration.
- Multivariable logistic regression assessed the association of each NETosis biomarker with the binary 12-month outcome mRS ≥ 3 vs. mRS < 3 in ICH patients. Odds ratios (OR) and 95% CIs are reported per doubling (per log2 unit) of the biomarker concentration.
- Multivariable Cox proportional hazards regression assessed the association of each NETosis biomarker, as a log2-transformed continuous variable and as a Q4vs. Q1–3 categorical variable, with all-cause death over 12 months. The date of blood collection served as the baseline for time-to-event analyses, and patients were censored at the date of last follow-up. Hazard ratios (HR) and 95% CIs are reported per doubling (continuous) or per Q4vs. Q1–3 contrast (categorical). The Q1–3 vs. Q4 cutoff (75th percentile) followed the convention used in established NET biomarker cohort studies (33). Kaplan–Meier curves were drawn for the same Q4 vs. Q1–3 strata and compared with the log-rank test.

The linear regressions of admission severity (Table 1) were first adjusted for time from hemorrhage to blood collection and then additionally for age, sex, current smoking, hypertension, prior cerebrovascular disease, and diabetes mellitus. In the prognostic analyses of 12-month mRS ≥ 3 and 12-month death in ICH patients (Tables 2–4), Model 1 was adjusted for time from hemorrhage to blood collection, and Model 2 was further adjusted for age, sex, current smoking, hypertension, prior cerebrovascular disease, diabetes mellitus, and surgical treatment. To probe whether the NETosis–outcome associations were independent of conventional severity scores, Model 2 was additionally extended with NIHSS, hematoma volume, or both.

**Table 1:** Linear regression of ICH severity on three NETosis biomarkers.

Variable	Time-adjusted		Multivariable-adjusted	
	*β* (95% CI)	*p*-value	*β* (95% CI)	*p*-value
Hematoma volume (mL)				
H3Cit	0.377 (0.263 to 0.490)	**<0.001**	0.291 (0.181 to 0.400)	**<0.001**
cfDNA	0.568 (0.299 to 0.836)	**<0.001**	0.279 (0.012 to 0.547)	**0.041**
Nucleosome	0.437 (0.244 to 0.631)	**<0.001**	0.214 (0.017 to 0.410)	**0.033**
NIHSS				
H3Cit	0.235 (0.180 to 0.290)	**<0.001**	0.206 (0.151 to 0.262)	**<0.001**
cfDNA	0.202 (0.066 to 0.339)	**0.004**	0.077 (−0.064 to 0.219)	0.282
Nucleosome	0.168 (0.070 to 0.266)	**<0.001**	0.104 (0.000 to 0.207)	**0.049**
GCS				
H3Cit	−0.067 (−0.086 to −0.048)	**<0.001**	−0.060 (−0.079 to −0.040)	**<0.001**
cfDNA	−0.030 (−0.076 to 0.016)	0.205	0.005 (−0.044 to 0.053)	0.854
Nucleosome	−0.036 (−0.070 to −0.003)	**0.033**	−0.017 (−0.052 to 0.019)	0.354

ICH severity variables (hematoma volume, NIHSS, and GCS) were square-root-transformed before linear regression. NETosis biomarkers were log₂-transformed. Time-adjusted: adjusted for time from hemorrhage to blood collection. Multivariable-adjusted: adjusted for time from hemorrhage to blood collection, age, sex, current smoking, hypertension, prior cerebrovascular disease, and diabetes mellitus. Bold *p*-values indicate *p* < 0.05.

**Table 2:** Logistic regression of poor functional outcome at 12 months (mRS ≥ 3) on three NETosis biomarkers (all ICH patients, *n* = 315).

Biomarker	Unit	Model	OR (95% CI)	*p*-value
H3Cit	per log₂ unit	Model 1	1.688 (1.491–1.933)	**<0.001**
		Model 2	1.683 (1.469–1.953)	**<0.001**
		Model 2 + NIHSS	1.592 (1.383–1.854)	**<0.001**
		Model 2 + hematoma volume	1.635 (1.423–1.901)	**<0.001**
		Model 2 + NIHSS + hematoma volume	1.577 (1.368–1.838)	**<0.001**
cfDNA	per log₂ unit	Model 1	1.924 (1.514–2.482)	**<0.001**
		Model 2	1.661 (1.269–2.202)	**<0.001**
		Model 2 + NIHSS	1.710 (1.281–2.315)	**<0.001**
		Model 2 + hematoma volume	1.653 (1.250–2.212)	**<0.001**
		Model 2 + NIHSS + hematoma volume	1.687 (1.259–2.291)	**<0.001**
Nucleosome	per log₂ unit	Model 1	1.747 (1.456–2.121)	**<0.001**
		Model 2	1.647 (1.336–2.056)	**<0.001**
		Model 2 + NIHSS	1.611 (1.296–2.029)	**<0.001**
		Model 2 + hematoma volume	1.668 (1.340–2.106)	**<0.001**
		Model 2 + NIHSS + hematoma volume	1.622 (1.297–2.057)	**<0.001**

Outcome: poor functional outcome at 12 months (mRS ≥ 3), analyzed in all ICH patients. NETosis biomarkers were log₂-transformed. Model 1: adjusted for time from hemorrhagic to blood collection. Model 2: Model 1 + age, sex, current smoking, hypertension, prior cerebrovascular disease, diabetes mellitus, and surgical treatment. Model 2 + NIHSS / + hematoma volume / + NIHSS + hematoma volume: Model 2 additionally adjusted for the indicated clinical severity covariates (raw scale). Bold *p*-values indicate *p* < 0.05.

**Table 3:** Cox regression of all-cause mortality at 12 months on three NETosis biomarkers (continuous).

Biomarker	Unit	Model	HR (95% CI)	*p*-value
H3Cit	per log₂ unit	Model 1	1.475 (1.325–1.640)	**<0.001**
		Model 2	1.422 (1.272–1.591)	**<0.001**
		Model 2 + NIHSS	1.372 (1.219–1.545)	**<0.001**
		Model 2 + hematoma volume	1.381 (1.233–1.546)	**<0.001**
		Model 2 + NIHSS + hematoma volume	1.358 (1.207–1.528)	**<0.001**
cfDNA	per log₂ unit	Model 1	1.855 (1.488–2.314)	**<0.001**
		Model 2	1.566 (1.243–1.972)	**<0.001**
		Model 2 + NIHSS	1.639 (1.303–2.062)	**<0.001**
		Model 2 + hematoma volume	1.532 (1.216–1.930)	**<0.001**
		Model 2 + NIHSS + hematoma volume	1.619 (1.287–2.038)	**<0.001**
Nucleosome	per log₂ unit	Model 1	1.590 (1.348–1.876)	**<0.001**
		Model 2	1.551 (1.295–1.857)	**<0.001**
		Model 2 + NIHSS	1.537 (1.281–1.845)	**<0.001**
		Model 2 + hematoma volume	1.562 (1.305–1.869)	**<0.001**
		Model 2 + NIHSS + hematoma volume	1.568 (1.310–1.877)	**<0.001**

NETosis biomarkers were log₂-transformed. Model 1: adjusted for time from hemorrhagic to blood collection. Model 2: Model 1 + age, sex, current smoking, hypertension, prior cerebrovascular disease, diabetes mellitus, and surgical treatment. Model 2 + NIHSS / + hematoma volume / + NIHSS + hematoma volume: Model 2 additionally adjusted for the indicated clinical severity covariates (raw scale). Bold *p*-values indicate *p* < 0.05.

**Table 4:** Cox regression of all-cause mortality at 12 months on three NETosis biomarkers (categorical, Q4 vs. Q1-3).

Biomarker	Unit	Model	HR (95% CI)	*p*-value
H3Cit	Q4 vs. Q1-3	Model 1	3.887 (2.437–6.201)	**<0.001**
		Model 2	2.987 (1.852–4.816)	**<0.001**
		Model 2 + NIHSS	2.204 (1.306–3.719)	**0.003**
		Model 2 + hematoma volume	2.585 (1.604–4.168)	**<0.001**
		Model 2 + NIHSS + hematoma volume	2.214 (1.319–3.715)	**0.003**
cfDNA	Q4 vs. Q1-3	Model 1	3.181 (2.000–5.058)	**<0.001**
		Model 2	2.366 (1.456–3.846)	**<0.001**
		Model 2 + NIHSS	2.697 (1.664–4.373)	**<0.001**
		Model 2 + hematoma volume	2.263 (1.395–3.672)	**<0.001**
		Model 2 + NIHSS + hematoma volume	2.580 (1.589–4.190)	**<0.001**
Nucleosome	Q4 vs. Q1-3	Model 1	2.346 (1.467–3.752)	**<0.001**
		Model 2	2.547 (1.550–4.184)	**<0.001**
		Model 2 + NIHSS	2.508 (1.532–4.107)	**<0.001**
		Model 2 + hematoma volume	2.564 (1.584–4.150)	**<0.001**
		Model 2 + NIHSS + hematoma volume	2.588 (1.594–4.201)	**<0.001**

Q4 vs. Q1-3: each biomarker dichotomized at its 75th percentile (raw value) within the ICH cohort; HR represents Q4 (high) vs. Q1-3 (low). Model 1: adjusted for time from hemorrhagic to blood collection. Model 2: Model 1 + age, sex, current smoking, hypertension, prior cerebrovascular disease, diabetes mellitus, and surgical treatment. Model 2 + NIHSS/+ hematoma volume/+ NIHSS + hematoma volume: Model 2 additionally adjusted for the indicated clinical severity covariates (raw scale). Bold *p*-values indicate *p* < 0.05.

To quantify incremental predictive value, a pre-specified clinical baseline model was constructed comprising time from hemorrhage to blood collection, age, sex, current smoking, hypertension, prior cerebrovascular disease, diabetes mellitus, surgical treatment, NIHSS, and hematoma volume. Each NETosis biomarker (continuous, log2) was then added in turn. For the 12-month mRS ≥ 3 logistic outcome, the area under the receiver operating characteristic curve (AUC) was estimated using the pROC package (v1.19.0.1) and ΔAUC was tested with DeLong’s method; the continuous NRI and IDI were calculated using the PredictABEL package (v1.2.4). For the 12-month survival Cox outcome, Harrell’s C-index was estimated using the bootstrap method with 2000 replicates; continuous NRI and IDI were computed with the survIDINRI package (v1.1.2).

Healthy controls were recruited contemporaneously but were not matched to ICH patients and differed in age and sex. To address this, a 1:1 propensity-score-matched subset was constructed: The propensity score was the logit of the predicted probability from a logistic model of case status on age and sex, and matching was greedy nearest-neighbor without replacement, with a caliper of 0.2 standard deviations of the logit propensity score and exact matching on sex. Balance was assessed by standardized mean differences (SMDs), with SMD < 0.10 taken as adequate (Supplementary Tables S1, S2).

Two-sided *p*-values <0.05 were considered statistically significant. Analyses were performed in R version 4.2.0.

## 3 Results

### 3.1 Baseline characteristics and NETosis biomarker distributions

A total of 315 patients with first-ever spontaneous ICH and 160 community controls were included. ICH patients were older than controls (mean age 59.4 ± 11.1 vs. 46.1 ± 10.7 years, *p* < 0.001) and more often male [216 (68.6%) vs. 75 (46.9%), *p* < 0.001] (Table 5). Among ICH patients, 208 (66.0%) had hypertension, 125 (39.7%) were current smokers, 37 (11.7%) had prior cerebrovascular disease, and 34 (10.8%) had diabetes mellitus; 86 (27.3%) had intraventricular extension, and 130 (41.3%) received surgical treatment. The basal ganglia was the most common bleeding location (178, 56.5%), followed by lobar (16.8%), cerebellar (10.8%), thalamic (9.2%), and brainstem (6.7%) sites. The median hematoma volume was 13.6 mL (IQR 3.3–31.6); median admission GCS was 13 (IQR 11–15), and median admission NIHSS was 11 (IQR 6–15). The median onset-to-sampling interval was 13.0 h (IQR 6.0–20.0), with 70 patients (22.2%) sampled within 6 h of onset, 76 patients (24.1%) between 6 and 12 h, and 169 patients (53.7%) between 12 and 24 h (Table 5).

**Table 5:** Baseline characteristics of healthy controls and ICH patients, with three NETosis biomarkers.

Variable	Control (*n* = 160)	ICH (*n* = 315)	*p*-value	ICH without 12-month death (*n* = 243)	ICH with 12-month death (*n* = 72)	*p*-value
Age, years	46.1 ± 10.7	59.4 ± 11.1	**<0.001**	57.4 ± 11.0	66.0 ± 8.8	**<0.001**
Male, *n* (%)	75 (46.9)	216 (68.6)	**<0.001**	163 (67.1)	53 (73.6)	0.366
Onset-to-sampling time, h	\	13.0 (6.0–20.0)		13.0 (6.0–19.5)	12.5 (8.0–21.0)	0.450
Onset-to-sampling window, *n* (%)						0.815
<6 h	\	70 (22.2)		55 (22.6)	15 (20.8)	
6–12 h	\	76 (24.1)		60 (24.7)	16 (22.2)	
12–24 h	\	169 (53.7)		128 (52.7)	41 (56.9)	
Comorbidities and history						
Diabetes mellitus	\	34 (10.8)		27 (11.1)	7 (9.7)	0.907
Hypertension	\	208 (66.0)		156 (64.2)	52 (72.2)	0.262
Prior cerebrovascular disease	\	37 (11.7)		25 (10.3)	12 (16.7)	0.205
Ischemic heart disease	\	11 (3.5)		7 (2.9)	4 (5.6)	0.281
Cancer	\	4 (1.3)		4 (1.6)	0 (0.0)	0.577
Current smoking	\	125 (39.7)		94 (38.7)	31 (43.1)	0.597
Intraventricular hemorrhage	\	86 (27.3)		62 (25.5)	24 (33.3)	0.247
Surgical treatment	\	130 (41.3)		99 (40.7)	31 (43.1)	0.830
Glasgow Coma Scale	\	13 (11–15)		13 (11–15)	11 (9–14)	**<0.001**
NIHSS	\	11 (6–15)		10 (5–14)	15 (8–30)	**<0.001**
Hemorrhage location, *n* (%)						
Basal ganglia	\	178 (56.5)		138 (56.8)	40 (55.6)	
Lobar (hemisphere)	\	53 (16.8)		39 (16.0)	14 (19.4)	0.871
Cerebellum	\	34 (10.8)		26 (10.7)	8 (11.1)	
Thalamus	\	29 (9.2)		22 (9.1)	7 (9.7)	
Brain stem	\	21 (6.7)		18 (7.4)	3 (4.2)	
Hematoma volume, mL	\	13.6 (3.3–31.6)		9.8 (2.1–25.1)	29.4 (18.8–50.2)	**<0.001**
NETosis biomarkers						
H3Cit, ng/mL	3.9 (0.0–8.2)	37.4 (10.8–149.9)	**<0.001**	23.5 (8.7–70.0)	165.4 (70.5–375.8)	**<0.001**
cfDNA, ng/mL	203.1 (140.5–293.9)	568.6 (336.0–907.0)	**<0.001**	521.2 (316.4–763.5)	841.0 (518.2–1643.6)	**<0.001**
Nucleosome, MoM	0.958 (0.626–1.326)	3.688 (1.863–7.499)	**<0.001**	2.921 (1.647–6.018)	6.738 (3.121–12.768)	**<0.001**
Neutrophil count, 10^9/L	3.39 ± 1.03	9.41 ± 2.94	**<0.001**	9.29 ± 2.95	9.83 ± 2.88	0.273

Continuous variables: mean ± SD for age and neutrophil count; median (IQR) for hematoma volume, Glasgow Coma Scale, NIHSS, and NETosis biomarkers; tested by Mann–Whitney U. Categorical variables: *n* (%); tested by chi-square or Fisher’s exact test. *p*-values compare Control vs. all ICH and ICH with vs. without 12-month death. “\” = not available for controls; Bold *p*-values indicate *p* < 0.05.

When ICH patients were stratified by 12-month vital status, decedents (*n* = 72) were on average older than survivors (*n* = 243; 66.0 ± 8.8 vs. 57.4 ± 11.0 years, *p* < 0.001) but did not differ in sex, vascular risk-factor prevalence, intraventricular extension, surgical treatment, hemorrhage location, or onset-to-sampling interval. Decedents had a substantially larger hematoma volume [median 29.4 (18.8–50.2) vs. 9.8 (2.1–25.1) mL], a higher admission NIHSS [15 (8–30) vs. 10 (5–14)], and a lower admission GCS [11 (9–14) vs. 13 (11–15)] (all *p* < 0.001).

In 48 controls (30.0%), the H3Cit absorbance fell below the blank and was assigned a value of 0 ng/mL, whereas no ICH patient had a zero H3Cit value. All three plasma NETosis biomarkers were markedly higher in ICH patients than in controls: H3Cit 37.4 (10.8–149.9) vs. 3.9 (0.0–8.2) ng/mL, cfDNA 568.6 (336.0–907.0) vs. 203.1 (140.5–293.9) ng/mL, and nucleosomes 3.688 (1.863–7.499) vs. 0.958 (0.626–1.326) MoM (all *p* < 0.001) (Figure 1; Table 5). The absolute neutrophil count was similarly higher in ICH patients than in controls (9.41 ± 2.94 vs. 3.39 ± 1.03 × 10^9^/L, *p* < 0.001), consistent with an acute neutrophil mobilization response after ICH. Crucially, however, within the ICH cohort the absolute neutrophil count was very similar between 12-month survivors and decedents (9.29 ± 2.95 vs. 9.83 ± 2.88 × 10^9^/L, *p* = 0.273), whereas all three NETosis biomarkers were several-fold higher in decedents than in survivors [H3Cit 165.4 (70.5–375.8) vs. 23.5 (8.7–70.0) ng/mL; cfDNA 841.0 (518.2–1643.6) vs. 521.2 (316.4–763.5) ng/mL; nucleosomes 6.738 (3.121–12.768) vs. 2.921 (1.647–6.018) MoM; all *p* < 0.001] (Table 5). Within ICH patients, H3Cit, cfDNA, and nucleosomes were positively but mostly weakly correlated with each other and with the absolute neutrophil count: Spearman’s correlations of H3Cit with cfDNA, nucleosomes, and the neutrophil count were 0.19 (*p* < 0.001), 0.16 (*p* = 0.003), and 0.16 (*p* = 0.004), respectively; cfDNA was most strongly correlated with nucleosomes (*r* = 0.49, *p* < 0.001) and weakly with the neutrophil count (*r* = 0.18, *p* = 0.001); nucleosomes correlated weakly with the neutrophil count (*r* = 0.16, *p* = 0.003; Figures 2A–F).

**Figure 1:**
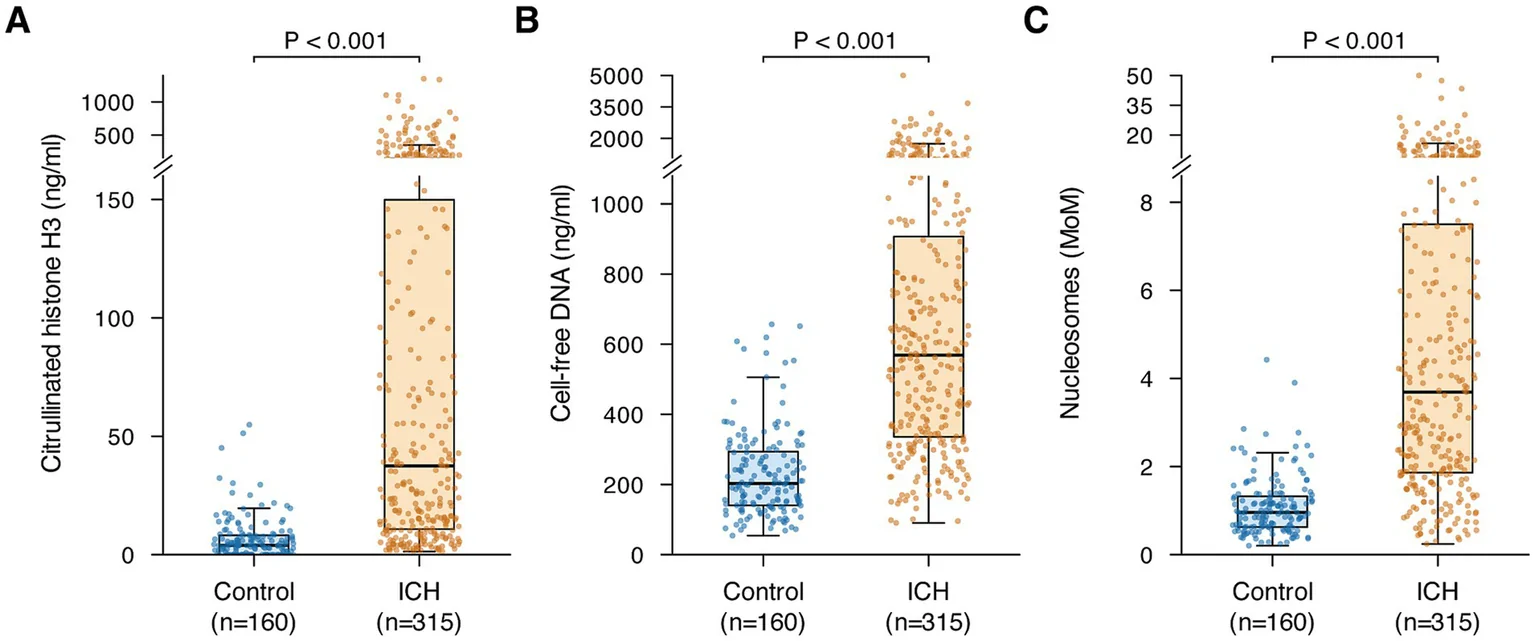
Plasma NETosis biomarkers in healthy controls (*n* = 160) and patients with spontaneous ICH (*n* = 315). (A) Citrullinated histone H3; (B) Cell-free DNA; (C) Nucleosomes. Boxes show the median and the interquartile range, and whiskers extend to 1.5 times the interquartile range. *p*-values are from the Mann–Whitney U test.

**Figure 2:**
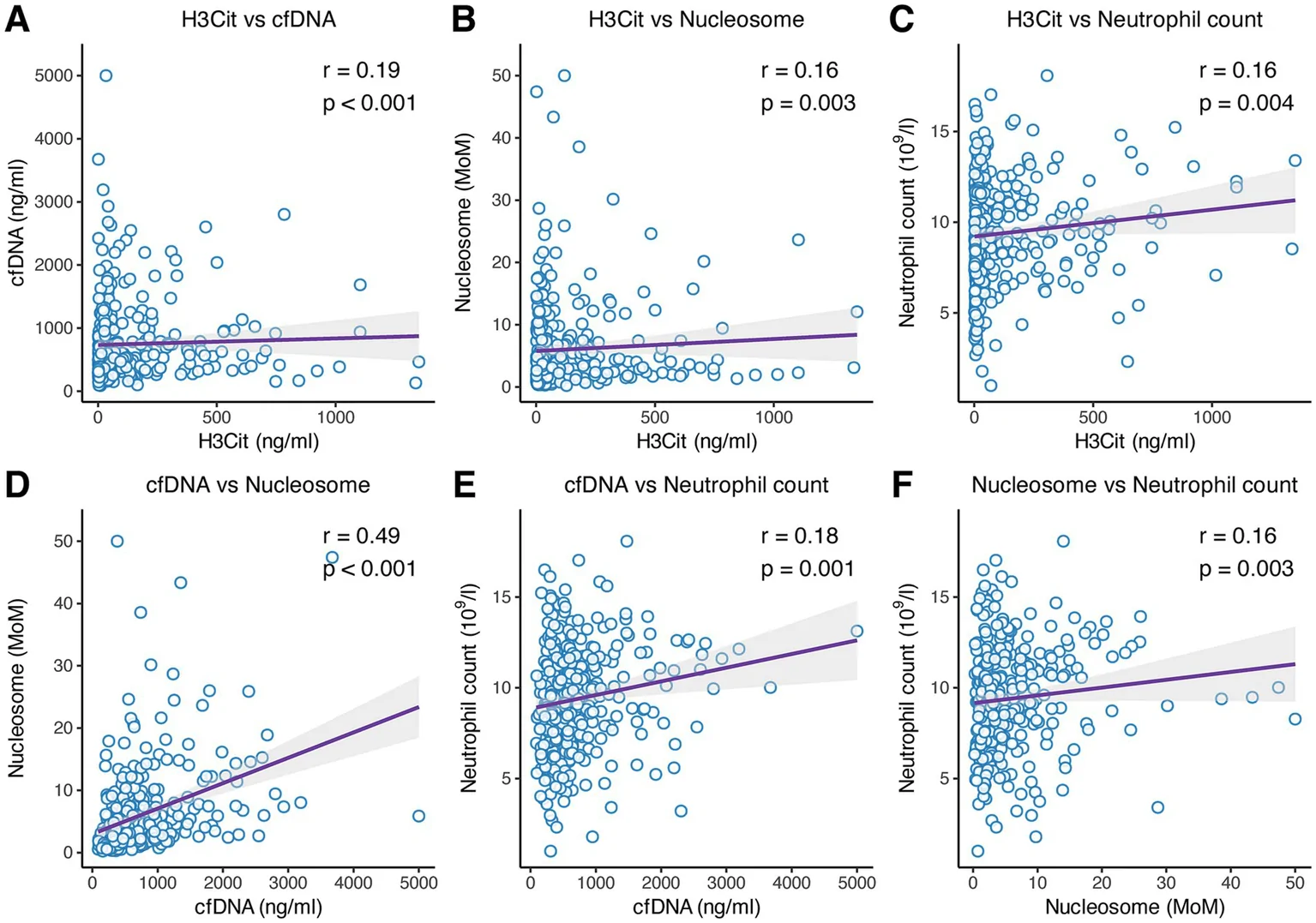
Pairwise scatter plots of plasma NETosis biomarkers and the absolute neutrophil count within the ICH cohort (*n* = 315). Each panel shows the Spearman correlation coefficient *r* and the corresponding *p*-value, with a linear regression line and 95% confidence band. **(A)**, H3Cit vs. cfDNA (*r* = 0.19, *p* < 0.001); **(B)** H3Cit vs. nucleosomes (*r* = 0.16, *p* = 0.003); **(C)** H3Cit vs. absolute neutrophil count (*r* = 0.16, *p* = 0.004); **(D)** cfDNA vs. nucleosomes (*r* = 0.49, *p* < 0.001); **(E)** (cfDNA vs. ab)solute neutr ophil count (*r* = 0.18, *p* = 0.001); **(F)** nucleosomes vs. absolute neutrophil count (*r* = 0.16, *p* = 0.003).

Because controls were younger and less often male than ICH patients, the between-group comparison was repeated in a propensity-score-matched subset. Matching on age and sex yielded 113 matched pairs with adequate balance for both covariates (SMD 0.087 for age and 0.000 for sex, both <0.10) (Supplementary Table S1). Within this matched subset, all three biomarkers remained markedly higher in ICH patients than in controls: H3Cit 21.8 (8.7–75.1) vs. 3.8 (0.0–7.6) ng/mL, cfDNA 462.3 (270.6–703.3) vs. 222.8 (149.0–304.4) ng/mL, and nucleosomes 2.761 (1.485–7.454) vs. 0.955 (0.627–1.312) MoM (all *p* < 0.001) (Supplementary Table S2).

Onset-to-sampling interval was examined as a potential source of variability in the measured concentrations. H3Cit was modestly higher in patients sampled latest [44.2 (14.6–200.8) ng/mL in the 12–24 h stratum vs. 25.9 (10.3–69.5) ng/mL in the <6 h stratum; Kruskal–Wallis *p* = 0.034], whereas cfDNA and nucleosomes showed no variation across strata (*p* = 0.518 and *p* = 0.497); correlations with sampling time as a continuous variable were weak for all three (Spearman’s *r* = 0.110, 0.004 and 0.089; Supplementary Table S3).

### 3.2 NETosis biomarkers and acute hemorrhage severity

Multivariable linear regression demonstrated that all three NETosis biomarkers were associated with greater hematoma volume and clinical severity at admission (Table 1). After adjustment for time from hemorrhage to blood collection, age, sex, current smoking, hypertension, prior cerebrovascular disease, and diabetes mellitus, each doubling of H3Cit was associated with a 0.291 unit increase in √hematoma volume (95% CI: 0.181 to 0.400, *p* < 0.001), a 0.206 unit increase in √NIHSS (95% CI: 0.151 to 0.262, *p* < 0.001), and a 0.060 unit decrease in √GCS (95% CI: −0.079 to −0.040, *p* < 0.001). Log2(cfDNA) was independently associated with √hematoma volume (*β* = 0.279, *p* = 0.041), but its associations with √NIHSS and √GCS were attenuated after adjustment. Log2(nucleosome) was independently associated with √hematoma volume (*β* = 0.214, *p* = 0.033) and √NIHSS (*β* = 0.104, *p* = 0.049). Across the three severity outcomes and three biomarkers, H3Cit thus showed the most consistent and statistically robust association with acute hemorrhage severity.

### 3.3 NETosis biomarkers and 12-month functional outcome

During 12-month follow-up, 72 patients (22.9%) died. An unfavorable 12-month functional outcome (mRS ≥ 3) was recorded in 123 of the 315 ICH patients (39.0%). Multivariable logistic regression showed that, per log2 unit, all three biomarkers were significantly associated with 12-month mRS ≥ 3 in every adjusted model (Table 2). After adjusting for time to blood collection alone (Model 1), the OR was 1.688 (95% CI: 1.491–1.933) for H3Cit, 1.924 (95% CI: 1.514–2.482) for cfDNA, and 1.747 (95% CI: 1.456–2.121) for nucleosomes (all *p* < 0.001). In the fully adjusted Model 2 + NIHSS + hematoma volume, all three retained an independent association with mRS ≥ 3: H3Cit OR 1.577 (95% CI: 1.368–1.838); cfDNA OR 1.687 (95% CI: 1.259–2.291); nucleosome OR 1.622 (95% CI: 1.297–2.057; all *p* < 0.001).

We next assessed the incremental value of each NETosis biomarker over a pre-specified clinical baseline logistic model containing time to blood collection, age, sex, current smoking, hypertension, prior cerebrovascular disease, diabetes, surgical treatment, NIHSS, and hematoma volume (Table 6; Figure 3). The baseline AUC for predicting mRS ≥ 3 was 0.814 (95% CI: 0.764–0.863). Adding log2(H3Cit) raised the AUC to 0.869 (95% CI: 0.830–0.909), a statistically significant improvement (ΔAUC: 0.055, 95% CI: 0.022–0.088, *p* < 0.001; continuous NRI: 0.803, *p* < 0.001; IDI: 0.108, *p* < 0.001). Adding cfDNA or nucleosomes yielded smaller AUC gains (0.015 and 0.021) that did not reach statistical significance (*p* = 0.150 and *p* = 0.070), although both significantly improved continuous NRI (0.379 and 0.479, both *p* < 0.001) and IDI (0.031, *p* = 0.002 and 0.042, *p* < 0.001).

**Table 6:** Incremental value of three NETosis biomarkers added to the baseline model for predicting poor functional outcome at 12 months (mRS ≥ 3).

Biomarker	Baseline AUC (95% CI)	With biomarker AUC (95% CI)	ΔAUC (95% CI)	*p*-value (ΔAUC)	NRI (95% CI)	*p*-value (NRI)	IDI (95% CI)	*p*-value (IDI)
H3Cit	0.814 (0.764 to 0.863)	0.869 (0.830 to 0.909)	0.055 (0.022 to 0.088)	**<0.001**	0.803 (0.595 to 1.011)	**<0.001**	0.108 (0.072 to 0.144)	**<0.001**
cfDNA	0.814 (0.764 to 0.863)	0.829 (0.782 to 0.875)	0.015 (−0.005 to 0.035)	0.150	0.379 (0.157 to 0.601)	**<0.001**	0.031 (0.011 to 0.051)	**0.002**
Nucleosome	0.814 (0.764 to 0.863)	0.835 (0.790 to 0.880)	0.021 (−0.002 to 0.045)	0.070	0.479 (0.259 to 0.699)	**<0.001**	0.042 (0.017 to 0.066)	**<0.001**

Outcome: poor functional outcome at 12 months (mRS ≥ 3), analyzed in all ICH patients. Baseline model: time from hemorrhagic to blood collection, age, sex, current smoking, hypertension, prior cerebrovascular disease, diabetes mellitus, surgical treatment, NIHSS, and hematoma volume. Each biomarker (log₂-transformed) is added on top of the baseline model. NRI, Continuous net reclassification improvement; IDI, Integrated discrimination improvement. Bold *p*-values indicate *p* < 0.05.

**Figure 3:**
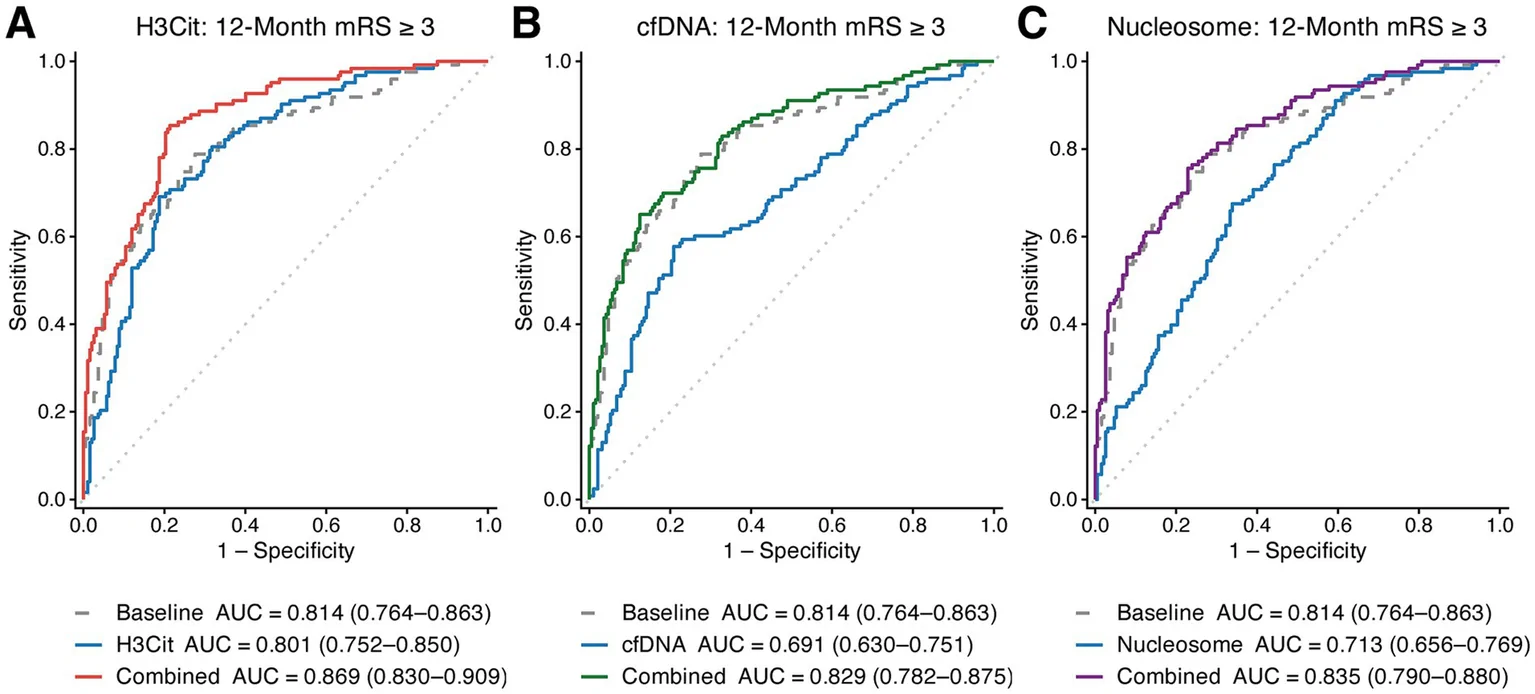
Receiver operating characteristic (ROC) curves for prediction of 12-month mRS ≥ 3 in all ICH patients (*n* = 315). **(A)**, H3Cit; **(B)**, cfDNA; **(C)**, nucleosomes. Each panel shows three ROC curves—the clinical baseline model, the biomarker alone, and the combined model—with corresponding AUCs and 95% CIs listed below. The clinical baseline model comprises time from hemorrhage to blood collection, age, sex, current smoking, hypertension, prior cerebrovascular disease, diabetes mellitus, surgical treatment, NIHSS, and hematoma volume.

### 3.4 NETosis biomarkers and 12-month survival

In Kaplan–Meier analyses with each biomarker dichotomized at the 75th percentile (Q4 vs. Q1–3), patients in Q4 of H3Cit, cfDNA, or nucleosomes had a markedly higher cumulative incidence of all-cause death over 12 months (log-rank *p* < 0.001 for all three; Figures 4A–C). Multivariable Cox regression confirmed these associations on a continuous scale (Table 3). In Model 2, the adjusted hazard ratio per log2 unit was 1.422 (95% CI: 1.272–1.591) for H3Cit, 1.566 (95% CI: 1.243–1.972) for cfDNA, and 1.551 (95% CI: 1.295–1.857) for nucleosomes (all *p* < 0.001). Importantly, these associations persisted after further adjustment for NIHSS, hematoma volume, or both: In the fully adjusted Model 2 + NIHSS + hematoma volume, the HR per log2 unit was 1.358 (95% CI: 1.207–1.528) for H3Cit, 1.619 (95% CI: 1.287–2.038) for cfDNA, and 1.568 (95% CI: 1.310–1.877) for nucleosomes (all *p* < 0.001).

**Figure 4:**
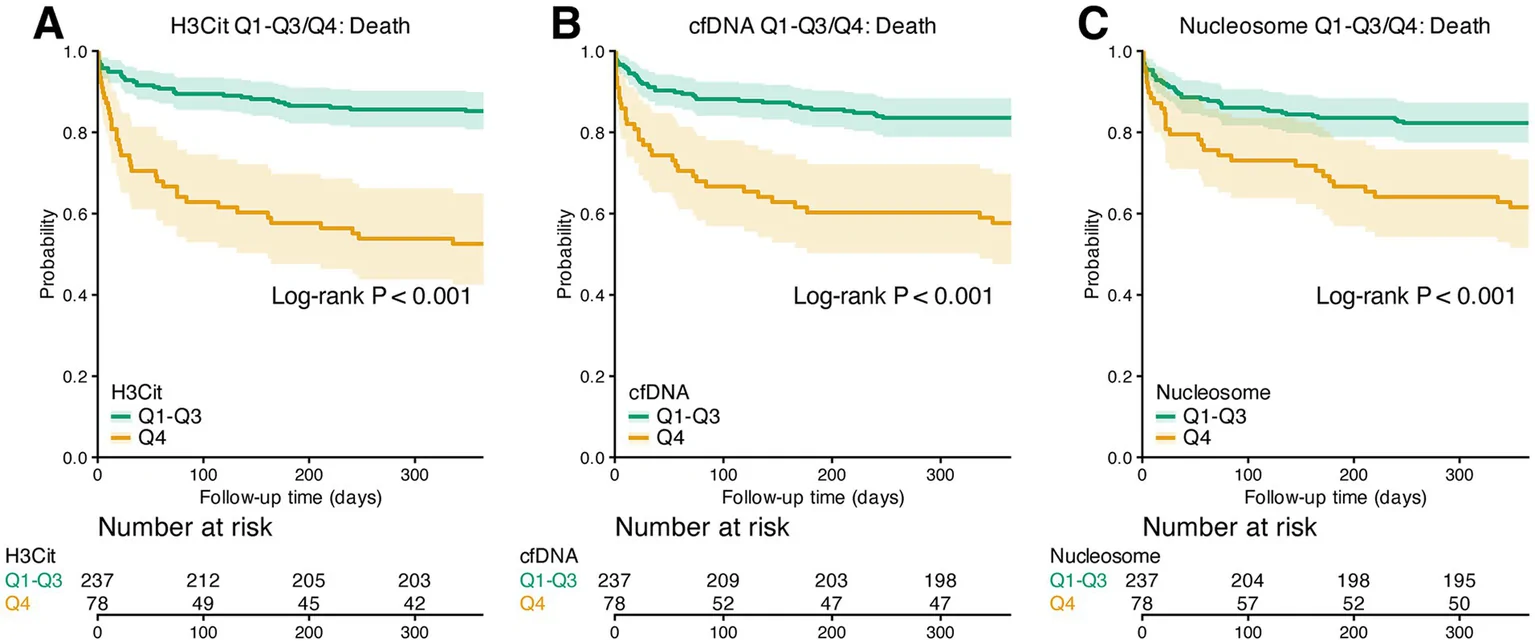
Kaplan–Meier survival curves and at-risk tables for 12-month all-cause death, stratified by quartile of each NETosis biomarker (Q4 vs. Q1–3). **(A)**, H3Cit; **(B)**, cfDNA; **(C)**, nucleosomes. Patients in the highest quartile (Q4) of each biomarker had significantly higher cumulative mortality than Q1–3 (log-rank *p* < 0.001 for all three).

When biomarkers were categorized as Q4 vs. Q1–3 (Table 4), unadjusted hazard ratios for Q4 vs. Q1–3 were 3.887 (95% CI: 2.437–6.201) for H3Cit, 3.181 (95% CI: 2.000–5.058) for cfDNA, and 2.346 (95% CI: 1.467–3.752) for nucleosomes (all *p* < 0.001). Associations remained robust after full adjustment (Model 2 + NIHSS + hematoma volume): H3Cit HR 2.214 (95% CI: 1.319–3.715, *p* = 0.003); cfDNA HR 2.580 (95% CI: 1.589–4.190, *p* < 0.001); nucleosomes HR 2.588 (95% CI: 1.594–4.201, *p* < 0.001).

Incremental survival prediction beyond a clinical baseline Cox model (the same 10 baseline covariates as in the logistic analysis) was evaluated using Harrell’s C-index, continuous NRI, and IDI (Table 7). The baseline C-index for 12-month death was 0.765. Adding H3Cit increased the C-index to 0.818 (ΔC-index: 0.052, 95% CI: 0.021–0.083, *p* < 0.001), with continuous NRI 0.760 (*p* < 0.001) and IDI 0.074 (*p* = 0.002). For nucleosomes, the gain in discrimination did not reach statistical significance (ΔC-index: 0.019, 95% CI: −0.000 to 0.049, *p* = 0.054), while continuous NRI (0.525, *p* < 0.001) and IDI (0.073, *p* < 0.001) were clearly improved. For cfDNA, the C-index change did not reach statistical significance (ΔC-index: 0.013, *p* = 0.170), but both continuous NRI (0.413, *p* = 0.028) and IDI (0.048, *p* = 0.017) were significantly improved. Across all metrics, H3Cit again showed the largest incremental gain.

**Table 7:** Incremental value of three NETosis biomarkers added to the baseline model for predicting all-cause mortality at 12 months.

Biomarker	Baseline C-index (95% CI)	With biomarker C-index (95% CI)	ΔC-index (95% CI)	*p*-value (ΔC)	NRI (95% CI)	*p*-value (NRI)	IDI (95% CI)	*p*-value (IDI)
H3Cit	0.765 (0.727 to 0.831)	0.818 (0.785 to 0.869)	0.052 (0.021 to 0.083)	**<0.001**	0.760 (0.408 to 1.020)	**<0.001**	0.074 (0.029 to 0.137)	**0.002**
cfDNA	0.765 (0.727 to 0.831)	0.778 (0.742 to 0.844)	0.013 (−0.005 to 0.041)	0.170	0.413 (0.065 to 0.729)	**0.028**	0.048 (0.010 to 0.097)	**0.017**
Nucleosome	0.765 (0.727 to 0.832)	0.784 (0.748 to 0.853)	0.019 (−0.000 to 0.049)	0.054	0.525 (0.302 to 0.815)	**<0.001**	0.073 (0.026 to 0.128)	**<0.001**

Outcome: all-cause death within 12 months, analyzed in the full ICH cohort. Baseline model: time from hemorrhagic to blood collection, age, sex, current smoking, hypertension, prior cerebrovascular disease, diabetes mellitus, surgical treatment, NIHSS, and hematoma volume. Each biomarker (log₂-transformed) is added on top of the baseline model. NRI, Continuous net reclassification improvement; IDI, Integrated discrimination improvement. Bold *p*-values indicate *p* < 0.05.

## 4 Discussion

In this cohort of 315 adults with first-ever spontaneous ICH, we systematically evaluated three circulating NETosis biomarkers measured within 24 h of onset—H3Cit, cfDNA, and nucleosomes—across multiple prognostic settings: Their elevation relative to community controls, their linear associations with admission hemorrhage severity (hematoma volume, NIHSS, and GCS), their prediction of 12-month mRS ≥ 3 in ICH patients, and their prediction of 12-month all-cause death, together with their incremental predictive value over an established clinical baseline model. All three biomarkers were markedly elevated in ICH patients and remained associated with adverse outcomes across adjusted models, but their performance was not uniform. H3Cit performed most consistently: After adjustment for demographics and vascular risk factors, it was independently associated with √hematoma volume, √NIHSS, and √GCS; in the fully adjusted Model 2 + NIHSS + hematoma volume, it remained an independent predictor of both 12-month mRS ≥ 3 and 12-month death; in incremental terms, it produced statistically significant improvements in ΔAUC/ΔC-index, continuous NRI, and IDI across both the logistic and Cox analyses, with AUC and C-index each improving by approximately 0.05. cfDNA and nucleosomes also independently predicted 12-month mRS ≥ 3 and death in the fully adjusted models and showed partial associations with acute hemorrhage burden, indicating residual prognostic value; their incremental contribution over the clinical baseline model was consistent in reclassification terms—continuous NRI and IDI were significantly improved for both biomarkers and both outcomes—but their gains in discrimination were modest and did not reach statistical significance for either ΔAUC or ΔC-index.

From a clinical perspective, these findings have several implications. First, they support a single, simple, biology-informed blood panel that can be obtained at admission and that captures information not fully encoded by NIHSS, GCS, or hematoma volume. All three biomarkers retained independent associations with 12-month mRS ≥ 3 and 12-month death after adjustment for these scores; H3Cit produced significant improvements in ΔAUC/ΔC-index, continuous NRI, and IDI in both the logistic and Cox analyses, whereas the incremental gains from nucleosomes and cfDNA—which we view as ancillary markers—were more modest. This complements previous study on other circulating ICH prognostic biomarkers such as NfL (15), SERPINA3 (16), IL-6 (17), and complement C1q (14, 18). Importantly, the recent ICH prognostic biomarker literature suggests that distinct circulating analytes capture different facets of post-ICH brain injury: Plasma NfL primarily reports on neuroaxonal damage and exhibits a biphasic time course, with the second peak in the subacute phase correlating with late white matter injury and cognitive decline (15); plasma SERPINA3 reports on astrocyte-driven and sustained neuroinflammation and is associated with chronic white matter hyperintensities and cognitive performance (16). Our panel adds an independent, neutrophil-derived innate-immune axis that, in the acute 24-h window, tracks the magnitude of the early bleeding insult and the ensuing neuroinflammatory cascade, providing information that is conceptually and temporally complementary to NfL and SERPINA3. Second, the strongest performance of H3Cit aligns with its biological specificity for PAD4-driven NETosis: In contrast to cfDNA and nucleosomes, which can rise with apoptosis, necrosis, and many systemic stressors (35, 36), H3Cit is largely restricted to neutrophil chromatin decondensation (33, 34), and is therefore a comparatively pure read-out of the immune mechanism we are interested in. Third, although the absolute neutrophil count was higher in ICH patients than in controls—as expected for an acute stress response—within the ICH cohort it did not distinguish 12-month decedents from survivors, whereas all three NETosis biomarkers were several-fold higher in decedents. This dissociation between bulk neutrophil count and NETosis activity reinforces the view that, beyond a generic neutrophilic response, it is the qualitative engagement of the NETosis program—rather than neutrophilia per se—that tracks with adverse ICH outcome, echoing the recent characterization of phenotypically distinct neutrophil subsets in ICH (27, 37). Combined with NfL and SERPINA3, NETosis indices may eventually form part of a complementary multi-marker panel covering neuronal, astrocytic, and neutrophilic injury after ICH (15, 16).

These findings should be read against the substantial and more mature literature on hemogram-derived immune indices in ICH. The NLR predicts mortality and poor functional outcome across 21 studies and more than 7,000 patients (20), the PWR improves survival nomograms in intensive-care ICH cohorts (21), and the systemic inflammation response index is independently associated with in-hospital infection and 3-month outcome (22). These indices are cheaper and more readily available than any research assay, and we do not propose that NETosis biomarkers should replace them. The distinction we would draw is one of level rather than of merit. All three are composite, non-specific indices of systemic inflammatory and immune activation: They quantify how many myeloid cells are present and in what proportion, and they rise whenever that compartment is mobilized, irrespective of which effector functions those cells go on to perform. H3Cit is tied instead to one defined molecular pathway—it quantifies whether those cells have executed PAD4-dependent chromatin decondensation, and thereby reflects the intensity of NETosis (38, 39). This distinction also has a translational aspect. The pathway that H3Cit reports is itself open to pharmacological interruption, which is not the case for a leucocyte ratio: PAD4 inhibitors such as GSK484 attenuate neuroinflammation and brain injury in models of hemorrhagic stroke (31, 40). Our data show that the intensity of this neutrophil program, measurable at the bedside within 24 h of onset, carries independent information about 12-month disability and death that established severity scores do not, which suggests that inhibition of NETosis may be a potential therapeutic avenue in ICH. NLR, PWR, and SIRI, by contrast, are non-specific markers of inflammatory activation, for which no comparably specific inhibitory strategy is available.

The biological plausibility of these associations is supported by an expanding mechanistic literature. NETs are extruded chromatin networks coated with citrullinated histones, neutrophil elastase, myeloperoxidase, and other granular proteins, and they amplify thromboinflammation through crosstalk with complement and coagulation (23, 24). After ICH, neutrophils accumulate in and around the hematoma, and intact NETs have been visualized in post-mortem human brain tissue and in animal models (25, 26). NETs propagate secondary brain injury in several ways relevant to the severity and outcome signals we observed. NETs promote perihematomal edema through ERK-mediated upregulation of MMP9 and AQP4, which would be expected to track with hematoma volume and clinical severity in the acute phase (28). NETs disrupt meningeal lymphatic drainage and exacerbate secondary hydrocephalus, plausibly contributing to long-term disability among survivors (29). NETs also engage in bidirectional crosstalk with microglia, sustaining chronic neuroinflammation that is increasingly recognized as a determinant of post-ICH outcome (9, 30). Consistent with these mechanisms, pre-clinical inhibition of NET formation attenuates brain injury and improves outcomes in models of ICH and ischemic stroke (31, 32), suggesting that the NETosis signal captured by H3Cit, cfDNA, and nucleosomes is not merely epiphenomenal but reports on a mechanistically actionable axis. In this light, our biomarker findings strengthen the rationale for testing NET-directed therapies in patients selected by high baseline NETosis activity and provide a clinically deployable readout that could enrich future neuroprotective trials.

This study has several limitations. First, it is a single-center cohort, and the findings, although internally robust to multiple sensitivity adjustments, require external validation across geographically and ethnically diverse ICH populations. Second, and most importantly for the interpretation of NETosis biology, no serial samples were obtained. NETosis after acute central nervous system injury is a dynamic immunothrombotic process, and our single measurement within 24 h of onset cannot describe its temporal trajectory. Stratification by onset-to-sampling interval showed only a modest gradient in H3Cit and none in cfDNA or nucleosomes, but comparing different patients sampled at different times is no substitute for repeated measurement within the same patient. Serial measurements would be needed to establish whether dynamic changes carry prognostic information beyond a single baseline value, in analogy to the biphasic profiles described for plasma NfL and SERPINA3 (15, 16); the absence of longitudinal sampling remains a principal limitation of this study. Third, none of the three indices is specific to ICH. H3Cit, cfDNA, and nucleosomes are all elevated in trauma, sepsis, malignancy, and metabolic and cardiovascular disease, and in systemic stress states more generally (35, 36), and can be influenced by concurrent infection or major systemic inflammation; residual confounding by occult comorbidity cannot be excluded despite our exclusion criteria. Relatedly, we did not measure complementary NETosis-related analytes such as neutrophil elastase–DNA or myeloperoxidase–DNA complexes, which could further refine assay specificity. Fourth, the H3Cit assay was built in-house using a polyclonal antibody (ab5103, Abcam). Because polyclonal reagents vary between lots in titer and epitope coverage, the absolute H3Cit concentrations reported here are not directly transferable to other laboratories, and standardization against a commercial, lot-controlled assay will be required before any clinical threshold can be proposed. Finally, while we report incremental improvement over a clinically rich baseline model, demonstration of clinical utility ultimately requires decision-impact studies.

## 5 Conclusion

In adults with first-ever spontaneous ICH, plasma H3Cit, cfDNA, and nucleosomes measured within 24 h of onset track admission hematoma volume and severity and independently predict 12-month functional outcome and survival beyond established clinical scores. H3Cit shows the most consistent incremental prognostic value, supporting its further development as a blood-based prognostic biomarker for ICH and as a candidate enrichment marker for NET-directed neuroprotective trials.

## Data Availability

The datasets presented in this article are not readily available because public sharing of the raw data is restricted due to patient privacy and confidentiality considerations, as well as ethical requirements related to the protection of personal health information. Requests to access the datasets should be directed to Lei Hui, drleihui@163.com.
