# Supplementary material for: Circulating NETosis biomarkers predict severity, 12-month functional outcome, and survival after spontaneous intracerebral hemorrhage
Source: Front Neurol. 2026 Sep 14;17:1901302. doi: 10.3389/fneur.2026.1901302 (PMC13616666; doi:10.3389/fneur.2026.1901302)
Supplement: Supplementary file 1 [file Table_1.DOCX]

**Supplementary Material**

**Supplementary Table S1 Covariate balance before and after 1:1 propensity-score matching of healthy controls to ICH patients**

| **Covariate** | **ICH before** | **Control before** | **SMD before** | **ICH after** | **Control after** | **SMD after** |
| --- | --- | --- | --- | --- | --- | --- |
| Age, years | 59.36 | 46.09 | 1.219 | 50.68 | 49.85 | **0.087** |
| Male sex, proportion | 0.69 | 0.47 | 0.449 | 0.54 | 0.54 | **0.000** |

*Propensity score: logit of the predicted probability from a logistic model of case status on age and sex. Greedy nearest-neighbour matching without replacement, caliper 0.2 standard deviations of the logit propensity score, with exact matching on sex. 113 pairs were formed from 315 ICH patients and 160 controls. SMD = standardised mean difference; SMD<0.10 is conventionally taken as adequate balance.*

**Supplementary Table S2 Biomarker concentrations in the propensity-score-matched subset (113 pairs)**

| **Biomarker** | **Matched controls (n=113)** | **Matched ICH patients (n=113)** | **P-value** |
| --- | --- | --- | --- |
| H3Cit, ng/mL | 3.8 (0.0-7.6) | 21.8 (8.7-75.1) | **<0.001** |
| cfDNA, ng/mL | 222.8 (149.0-304.4) | 462.3 (270.6-703.3) | **<0.001** |
| Nucleosome, MoM | 0.955 (0.627-1.312) | 2.761 (1.485-7.454) | **<0.001** |

*Median (interquartile range); Mann–Whitney U test.*

**Supplementary Table S3 Biomarker concentrations by onset-to-sampling window**

| **Biomarker** | **<6 h (n=70)** | **6-12 h (n=76)** | **12-24 h (n=169)** | **P (Kruskal–Wallis)** | **Spearman r vs sampling time** | **P** |
| --- | --- | --- | --- | --- | --- | --- |
| H3Cit, ng/mL | 25.9 (10.3-69.5) | 23.0 (8.9-126.8) | 44.2 (14.6-200.8) | **0.034** | 0.110 | 0.051 |
| cfDNA, ng/mL | 531.5 (342.1-788.2) | 571.3 (368.2-1008.2) | 586.4 (324.4-949.7) | 0.518 | 0.004 | 0.949 |
| Nucleosome, MoM | 3.214 (1.614-7.417) | 3.654 (1.980-7.431) | 3.910 (1.992-7.734) | 0.497 | 0.089 | 0.117 |

*Median (interquartile range). Only H3Cit varied across sampling windows, and only modestly; cfDNA and nucleosomes did not.*
